# Age-Related Differences in the Effect of Psychological Distress on Mortality: Type D Personality in Younger versus Older Patients with Cardiac Arrhythmias

**DOI:** 10.1155/2013/246035

**Published:** 2013-09-25

**Authors:** Johan Denollet, Fetene B. Tekle, Pepijn H. van der Voort, Marco Alings, Krista C. van den Broek

**Affiliations:** ^1^Center of Research on Psychology in Somatic Diseases (CoRPS), Department of Medical and Clinical Psychology, Tilburg University, P.O. Box 90153, 5000 LE Tilburg, The Netherlands; ^2^Department of Methodology and Statistics, Tilburg University, P.O. Box 90153, 5000 LE Tilburg, The Netherlands; ^3^Department of Cardiology, Catharina Hospital, Eindhoven, The Netherlands; ^4^Department of Cardiology, Amphia Hospital, Breda, The Netherlands; ^5^GGz Breburg, Tilburg, The Netherlands

## Abstract

*Background.* Mixed findings in biobehavioral research on heart disease may partly be attributed to age-related differences in the prognostic value of psychological distress. This study sought to test the hypothesis that Type D (*distressed*) personality contributes to an increased mortality risk following implantable cardioverter defibrillator (ICD) treatment in younger patients but not in older patients. *Methods.* The Type D Scale (DS14) was used to assess general psychological distress in 455 younger (≤70 y, *m* = 59.1) and 134 older (>70 y, *m* = 74.3) ICD patients. End points were all-cause mortality and cardiac death after a median follow-up of 3.2 years. *Results.* Older patients had more advanced heart failure and a higher mortality rate (*n* = 34/25%) than younger patients (*n* = 60/13%), *P* = 0.001. Cardiac resynchronization therapy (CRT), but not Type D personality, was associated with increased mortality in older patients. Among younger patients, however, Type D personality was associated with an adjusted hazard ratio = 1.91 (95% CI 1.09–3.34) and 2.26 (95% CI 1.16–4.41) for all-cause and cardiac mortality; other predictors were increasing age, CRT, appropriate shocks, ACE-inhibitors, and smoking. *Conclusion.* Type D personality was independently associated with all-cause and cardiac mortality in younger ICD patients but not in older patients. Cardiovascular research needs to further explore age-related differences in psychosocial risk.

## 1. Introduction

Beyond the age of 70 years, the risk of progressive heart failure [1, 2] and mortality [2, 3] sharply increases in cardiac patients. Age-related biological changes, such as decreased cardiomyocyte renewal capacity and increased cardiac dysfunction, contribute to poor health in older cardiac patients [1]. There are also important age-related differences in the clinical profile and outcome of patients who are at risk for life-threatening cardiac arrhythmias [4–8]. Today, implantable cardioverter defibrillator (ICD) therapy is the first-line treatment for the prevention of sudden cardiac death in these patients [9]. When a significant arrhythmia is detected, the ICD restores a normal heart rhythm by providing antitachycardia pacing or, eventually, a shock. However, advanced heart failure [4–6] and comorbid conditions [4, 5, 7] are common in older patients and may attenuate the survival benefit of ICD treatment [4]. 

Psychological distress has been associated with a higher risk of ventricular arrhythmias and mortality in some [10–12] but not all [13, 14] studies of ICD patients, suggesting that other variables may moderate this association. For example, differences in the nature and timing of depression have also contributed to mixed findings on depression and heart disease [15]. To resolve the uncertainty about a causal link between mind and heart [16], we need to identify factors that contribute to mixed findings on psychological distress in cardiac patients. Critically, these mixed findings may partly be attributed to age-related differences in distress.

The influence of psychological distress on cardiovascular outcomes may be more potent in younger than in older patients [17–19]. Psychological distress is particularly pronounced in younger ICD patients [20, 21] and may induce changes in the autonomous nervous system that have been implicated in the onset of ventricular arrhythmia [22–26]. Type D (distressed) personality is a propensity to general distress (i.e., the combination of negative affectivity and social inhibition) that may also contribute to cardiovascular outcomes [27, 28], including poor health [29] and decreased survival [30, 31] in patients with an ICD. However, Type D was not associated with prognosis in patients with heart failure [32] and little is known about age-related differences in the effect of Type D among patients with cardiac arrhythmia. 

The development of novel risk stratification strategies is needed to improve outcomes in patients with an ICD [9], and a scientific statement from the American Heart Association concluded that psychosocial factors such as Type D personality may be involved in clinical events following ICD treatment [33]. Previously, we reported that Type D and device shocks were associated with an increased mortality risk in a cohort study of 589 ICD patients [31]. Yet, cardiac disease is a complex condition, and a better understanding of the associations between distress and prognosis also involves subgroup analyses [34] that reveal at what ages or in what clinical subgroups such associations hold [19, 35]. Others have used 70 years as split-point to examine the outcome of ICD treatment in different age subgroups [7] and we showed that depression predicted poor prognosis in cardiac patients younger than 70 years but not in older patients [18]. Regarding clinical subgroups, little is known about the combined effect of shocks and psychological distress on the outcome of ICD treatment.

Therefore, we examined the age-dependent role of shocks and Type D personality and tested the hypothesis that Type D personality contributes to an increased mortality risk in younger ICD patients in particular. In addition, we explored whether the combination of shocks and Type D personality would involve an increase in risk among younger patients.

## 2. Methods

### 2.1. Patient Sample

This study reports on preplanned analysis of age-related differences in an observational study of psychosocial stress among 589 patients living with an ICD because of a high risk of life-threatening cardiac arrhythmia. Details of this study have been described elsewhere [31]. Patients all had a first ICD implanted in the Amphia Hospital, Breda, or Catharina Hospital, Eindhoven, The Netherlands, between May 2003 and February 2009. The age ranged from 18 to 80 years; patients with cognitive impairment (e.g., dementia) and psychiatric disorders other than affective disorder were excluded. The study was conducted in accordance with the Helsinki Declaration, and all patients provided written informed consent. The study was approved by the Medical Ethics Committees of both participating hospitals. 

### 2.2. Type D Personality

In order to assess Type D personality, all patients completed the 14-item Type D Scale (DS14) [36] at the time of implantation [31]. Type D refers to the combination of the tendency to experience negative emotions (negative affectivity) and the tendency to inhibit self-expression in social interaction (social inhibition) that has been associated with an increased vulnerability for psychological distress and poor cardiovascular outcomes [27]. The 7-item negative affectivity and social inhibition subscales of the DS14 are internally consistent (Cronbach's *α* = 0.88 and 0.86) and stable over time [36]. Patients scoring above the standardized cut-off ≥10 on both subscales are classified as having a Type D personality.

### 2.3. Younger versus Older Age

To examine the age-related differences in clinical profile, survival, and prognostic markers, patients were stratified by age into younger and older age groups. In analogy with previous ICD research, patients aged ≤70 years were considered to be relatively younger compared to patients aged >70 years [7]. In addition, previous research on Type D personality [27] largely focused on relatively younger patients aged <70 years or <75 years, with the mean age ranging between 55 and 57 years. Hence, ≤70 year was also used as a cut-off in the present study to test the hypothesis that the tendency to experience psychological distress might primarily contribute to poor survival in younger rather than older patients [18, 19].

### 2.4. Endpoints

The first endpoint was all-cause death. Because some patients do not benefit from ICD therapy because of competing noncardiac death risks [9], we also examined the association of Type D with cardiac death as a more rigorous endpoint of cardiac prognosis. As discussed previously, the endpoints in this study were derived from the medical records, and the cause of death was discussed with the treating cardiologist or general practitioner [31].

### 2.5. Shocks and Other Statistical Covariates

Device shocks have been related to an increased mortality risk in patients with an ICD [4, 5] and were included in all multivariable analyses. Electrophysiologists judged the nature of shocks on the basis of device interrogation; ICD shocks were considered to be inappropriate if they were triggered by abnormal sensing or by nonventricular arrhythmias and to be appropriate if they were triggered by ventricular tachycardia or by ventricular fibrillation. Some patients were treated with the combination of an ICD with cardiac resynchronization therapy (CRT-D); because CRT-D further improves the clinical course of heart failure [37], we controlled for this treatment variable in multivariable models. Other statistical covariates included left ventricular ejection fraction (LVEF) ≤35%, primary versus secondary indication for ICD treatment, coronary artery disease (CAD), beta-blockers, ACE-inhibitors, diabetes mellitus, smoking, gender, and marital status (having a partner versus having no partner).

### 2.6. Statistical Analyses

Clinical characteristics of the sample stratified by age were examined with chi-square tests for categorical variables and *t*-tests for independent samples for continuous variables. Multivariable Cox regression analyses were used to examine the hypothesis that Type D may play an independent prognostic role in younger ICD patients (≤70 years). Separate multivariable regression models were construed for both all-cause and cardiac-related death. A chi-square test was used to explore the combined effect of shocks and Type D personality in younger patients. All tests were two-tailed and a *P* value < 0.05 was used to indicate statistical significance. All analyses were performed in PASW Statistics 17 for Windows.

## 3. Results

### 3.1. Clinical Characteristics of Younger versus Older Patients

The mean age of the ICD patients was 62.6 years; 455 patients were aged ≤70 years and 134 aged >70, with the first subgroup being on average 15 years younger than the second subgroup (59 versus 74 years). This stratification by age revealed a different clinical profile; that is, older patients were more likely to have advanced heart failure, as indicated by a higher prevalence of left ventricular dysfunction and CRT treatment, than younger patients (Table 1). After a median follow-up of 3.2 (range 0.8–6.5) years, there were 94 (16%) ICD patients who had died from all causes and 67 (11%) patients who had died from cardiac causes [31]. The rate of both all-cause death and cardiac death was approximately 2 times higher in older ICD patients compared with younger ICD patients (*P* ≤ 0.003; Table 1). The mean age at the time of death was 77 years in older patients and 64 years in younger patients.

### 3.2. Prognosis in Older versus Younger Patients

**Figure 1:**
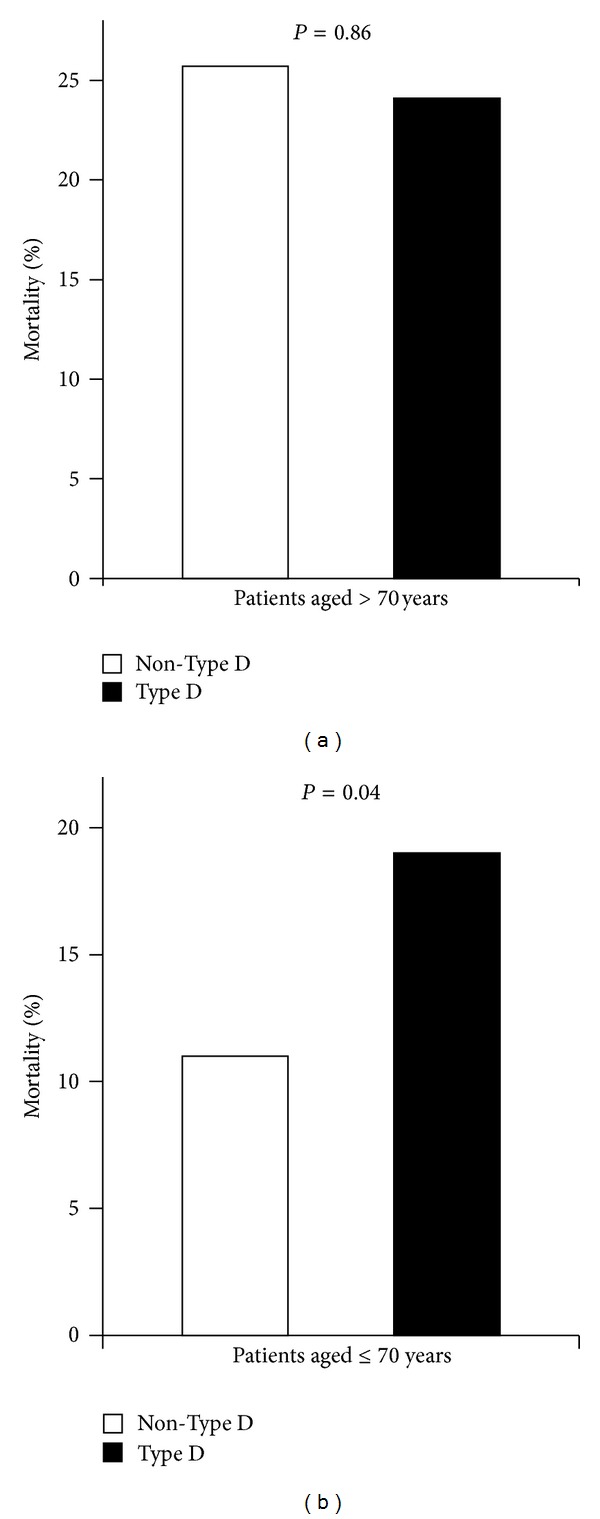
Association of Type D personality with all-cause mortality in older (a) and younger (b) ICD patients.

To examine the age-dependent importance of prognostic markers and to test the hypothesis that psychological distress contributes to the incidence of death in younger ICD patients, we analyzed the predictors of mortality separately in older and younger patients. Among older patients (>70 years), Type D personality was not associated with mortality (Figure 1(a)). CRT was the only independent predictor of all-cause mortality in older patients (HR = 2.51, 95% CI 1.05–5.97, *P* = 0.038); there was also a trend for diabetes (HR = 2.14, 95% CI 0.95–4.81, *P* = 0.06) but neither for appropriate shocks (*P* = 0.42) nor other covariates (*P* > 0.10).

Among younger ICD patients (≤70 years), Type D personality was associated with an increased risk of death from all causes (Figure 1(b)). Type D patients had an adjusted HR = 1.91 (95% CI 1.09–3.34) for all-cause mortality; other significant predictors were increasing age, CRT, appropriate shocks, (lack of) ACE-inhibitors, and smoking (Table 2). 

### 3.3. Type D Personality and Cardiac Death in Younger Patients

**Figure 2:**
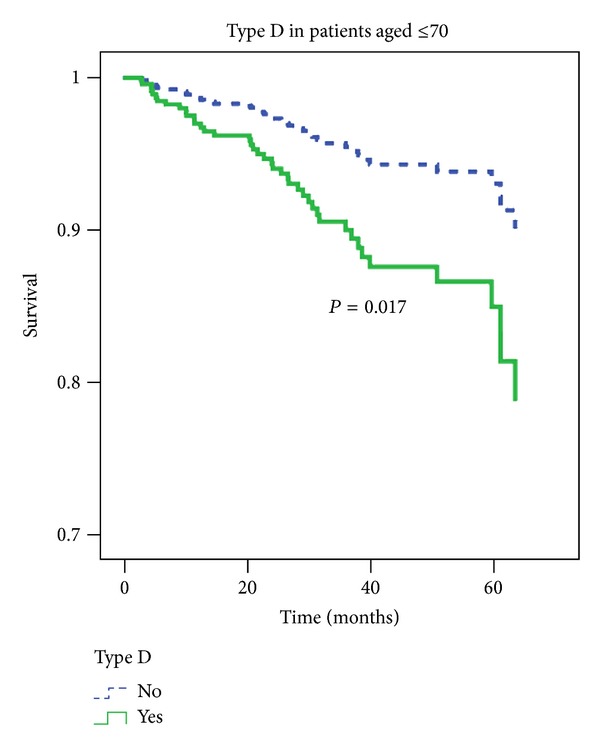
Cardiac survival of younger ICD patients (*N* = 455), stratified by Type D personality. Cardiac death (*N* = 42) coded as 1. Multivariable analyses, adjusted for cardiac covariates and noncardiac covariates.

Given the cardiovascular effects of distress [10, 11, 25, 26], cardiac death was included as a second endpoint in this study. Type D personality was associated with an increased risk of cardiac death in younger ICD patients (Figure 2). In a multivariable Cox regression model, Type D personality was retained as an independent predictor of cardiac death in younger ICD patients (HR = 2.26, 95% CI 1.16–4.41, *P* = 0.017), after adjustment for CRT (HR = 3.08, 95% CI 1.53–6.22, *P* = 0.002) and appropriate shocks (HR = 3.95, 95% CI 1.75–8.91, *P* = 0.001). There was also a trend for increasing age (*P* = 0.06) and smoking (*P* = 0.053) in these patients. 

**Figure 3:**
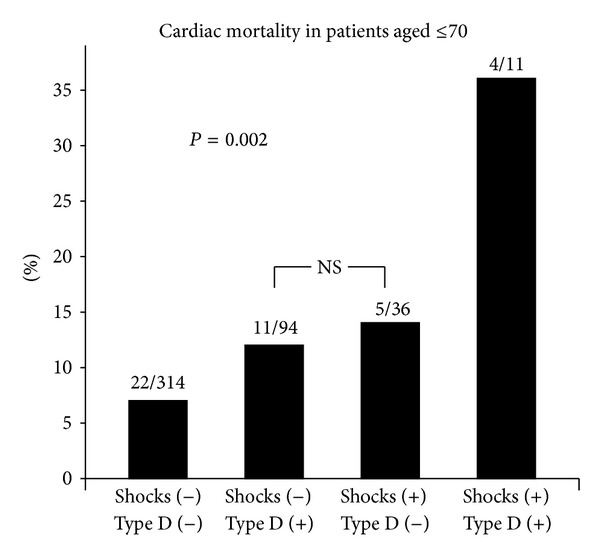
Percentage of younger ICD patients who died of cardiac causes (*N* = 42), stratified by appropriate shocks and Type D personality. NS = not significant; other groups are significantly different from each other. Shocks (−) = no appropriate shocks during follow-up; Shocks (+) = one or more appropriate shocks during follow-up; Type D (−) = no Type D personality; Type D (+) = Type D personality. Number of patients who died of cardiac causes and total number of patients within each risk subgroup are presented on top of each bar.

Finally, cardiac death was examined in 4 groups of younger patients, stratified by Type D and shocks (Figure 3). Patients with Type D or shocks only had a similar risk of cardiac death. Risk of cardiac death was highest in Type D patients who received a shock (36%), followed by patients with one risk marker only (12%); patients with no marker had the lowest risk. 

## 4. Discussion

ICD shocks have been related to an increased mortality risk [4, 5], but a scientific statement from the American Heart Association also highlighted the need to improve the identification and care of psychosocial factors, such as Type D personality, in patients with an ICD [33]. We previously reported that Type D may have detrimental consequences for the outcome of ICD treatment in this cohort of patients with life-threatening cardiac arrhythmias [31] and that Type D personality may be associated with ventricular arrhythmias in ICD patients [38]. Previous research in another cohort of patients with an ICD also showed that Type D was significantly associated with an increased risk of emotional distress [29] and mortality [30]. 

However, patients who are older already have an increased mortality risk due to biological aging of the cardiovascular and other organic systems [1–3], which limits the incremental prognostic value of psychosocial distress. Accordingly, the present analyses showed that Type D personality was associated with mortality in younger but not older ICD patients. Age-related differences in prognosis may result from different mechanisms of disease. Among older patients, CRT and diabetes, but not shocks or Type D, were associated with mortality. Evidence suggests that age-related biological changes may contribute to adverse clinical outcomes [1, 6]. Cardiac dysfunction is particularly pronounced in older patients due to decreased cardiomyocyte renewal capacity and increased apoptosis [1]. In addition, progressive heart failure is a common cause of death in ICD patients aged >70 years [4–6]. In this ICD cohort, older patients were also more likely to have a CRT-D and left ventricular dysfunction than younger patients, which may partly explain their increased mortality risk.

In our ICD cohort, shocks and Type D personality were associated with all-cause and cardiac death in younger patients. Although younger ICD patients had a better survival rate than older patients, a substantial number of younger patients had a poor prognosis. The mean age at the time of death was 64 years in the 60 younger patients who died (and 77 years in the 34 older nonsurvivors), which highlights the high-risk profile of this subgroup. Chronic psychological distress may explain why some younger patients were at an increased risk of premature death. This age-dependent effect of distress may reflect selective survival [17]; that is, Type D patients who die at an earlier age may be more vulnerable to stress-related cardiac dysfunction and arrhythmia as compared to Type D patients who survive to old age. 

The adverse effects of Type D in younger patients might be explained by the consequences of chronic psychosocial distress [27]. These may include direct biological consequences. Among other things, Type D personality has been associated with an increased prevalence of ventricular arrhythmia in healthy individuals [39] and in patients with an ICD [38] and with decreased heart rate recovery after exercise in patients with chronic heart failure [40]. There also may be indirect behavioral consequences that have an adverse effect on health. For example, Type D has been related to poor adherence to continuous positive airway pressure therapy in patients with obstructive sleep apnea [41], which, in turn, may cause disturbed cardiac repolarization that can induce cardiac arrhythmias in these patients [42]. These explanations are still hypothetical, but further research is warranted to examine whether these pathways of disease associated with Type D personality are age-dependent. 

This study has a number of limitations [31]. First, we did not have information on changes in disease severity that may have occurred during follow-up. Second, 35% of patients with CRT had a poor LVEF compared to only 2% of patients without CRT (*P* < 0.001). This overlap in clinical covariates may explain why CRT, but not LVEF, was an independent predictor of mortality in the multivariable regression model. Third, other markers of disease severity or somatic comorbidity that were not included may also have affected survival. Fourth, more research is needed to examine clinical implications. Initial evidence suggests that age might moderate the effect of behavioral intervention: two trials found that younger ICD patients are more likely to improve in psychological distress than older patients [43, 44]. This may also apply to modification of emotion regulation strategies in Type D patients [45]. Strengths of this study are the large sample of patients who are at high risk of arrhythmia, the prospective design of the study, and the standardized assessment of Type D personality.

Using Type D personality and ICD treatment of life-threatening arrhythmias as a paradigm, the present study highlights the importance of age-dependent differences in psychosocial risk. Other reports also showed that the link between heart disease and hostility or depression was more pronounced in young individuals [17, 18]. Consistent with these reports, we observed that younger ICD patients may have an increased mortality risk due to psychosocial distress. In this age group, the risk of cardiac death was the highest in Type D patients who received a shock. Hence, these findings indicate that clinical subgroup analyses may further improve our understanding of the associations between psychosocial distress and prognosis [34, 35]. However, the prognostic value of distress in older patients is limited due to cardiovascular and biological aging. In our study, cardiac resynchronization therapy, ventricular systolic dysfunction, and diabetes were more common and predicted mortality in older ICD patients. In conclusion, health professionals should be aware of age-related individual differences in clinical risk, and research needs to further examine the extent to which the adverse effect of psychosocial factors on the incidence and progression of heart disease is dependent on age.

## Figures and Tables

**Table 1:** Characteristics of study population, stratified by age ≤/> 70 years.

Characteristics	Age ≤ 70	Age > 70	*P* value
	*N* = 455	*N* = 134	
Demographic			
Age (years; mean ± SD)	59.1 ± 8.9	74.3 ± 2.5	**0.0001**
No partner	11% (49)	22% (29)	**0.001**
Male gender	81% (368)	81% (108)	0.94
Personality			
Type D personality	23% (105)	22% (29)	0.73
Mortality			
All-cause death	13% (60)	25% (34)	**0.001**
Cardiac death	9% (42)	19% (25)	**0.003**
ICD treatment			
LVEF ≤ 35%	80% (364)	92% (123)	**0.002**
CRT therapy	26% (120)	41% (55)	**0.001**
Primary indication ICD	63% (289)	67% (90)	0.44
Appropriate shocks FU	10% (47)	10% (14)	0.97
Inappropriate shocks FU	4% (20)	6% (8)	0.45
Medical covariates			
Diagnosis of CAD	71% (325)	77% (103)	0.21
Beta-blocker therapy	82% (375)	79% (106)	0.38
ACE-inhibitor therapy	67% (306)	70% (94)	0.53
Diabetes	18% (82)	22% (29)	0.35
Smoking	20% (89)	13% (17)	0.07

CAD: coronary artery disease; CRT: cardiac resynchronization therapy; LVEF: left ventricular ejection fraction; SD: standard deviation.

**Table 2:** Independent predictors of death from all causes in ICD patients aged ≤70.*

	HR (95% CI)	*P* value
Predictor variables		
Type D personality	1.91 (1.09–3.34)	0.024
Age (years)	1.06 (1.02–1.10)	0.008
CRT	1.78 (0.99–3.27)	0.056
Appropriate shocks	3.50 (1.79–6.82)	0.0001
ACE-inhibitors	0.51 (0.30–0.87)	0.014
Smoking	1.85 (1.01–3.40)	0.049

*Values were calculated with the use of multivariable Cox regression analysis, including the nonsignificant covariates (gender, inappropriate shocks, left ventricular ejection fraction, secondary indication, coronary artery disease, beta-blockers, diabetes, and partner status).

CI: confidence interval; CRT: cardiac resynchronization therapy; HR: hazard ratio.
